# Formation mechanism of the (2 × 1) reconstruction of calcite (104)

**DOI:** 10.1038/s41598-025-95955-2

**Published:** 2025-04-08

**Authors:** Haojun Zhou, Yingquan Chen, Mingyue Ding, Xiaoliang Zhong

**Affiliations:** 1 School of Energy and Power Engineering, Huazhong University of Science and Technology, Wuhan, 430074 China; 2 School of Power and Mechanical Engineering, Wuhan University, Wuhan, 430072 China

**Keywords:** Chemistry, Materials science

## Abstract

Calcite has recently attracted extensive research interest in fields ranging from geoscience to carbon dioxide removal. Although much effort has been made to study the (2 × 1) reconstruction of the most stable (104) surface, the origin of this reconstruction remains unclear. Here, we carefully investigated the atomic and electronic structures of calcite (104) via density functional theory methods with van der Waals corrections. The results unambiguously show that the driving force for this reconstruction is the intrinsic demands of surface atoms to increase the coordination numbers. Upon reconstruction, calcite (104) forms four additional Ca‒O bonds per (2 × 1) unit cell. In addition, the phonon spectra indicate that both the unreconstructed and reconstructed surfaces are dynamically stable. Finally, by applying the climbing image nudged elastic band method, an energy barrier is predicted during the reconstruction. This work provides a full picture of the formation of calcite (104)-(2 × 1) reconstruction and can greatly advance the understanding of surface science for calcite.

**Supplementary Information:**

The online version contains supplementary material available at 10.1038/s41598-025-95955-2.

## Introduction

Calcium carbonate (CaCO_3_) is a common substance on Earth. It is the major constituent of limestone, marble, eggshells, and pearls. As the major polymorph of CaCO_3_ in nature^1^, calcite has been actively investigated in fields such as geoscience^2–4^, soil stabilization^5,6^, carbon dioxide removal^7^, and new material development^8,9^. The most stable face of calcite is the (104) surface, with the fewest Ca–O bonds broken upon surface formation^10^. This surface supports virtually all processes involving calcite^11^. It is known that surface reconstruction can have a profound impact on crystal growth, surface properties and the application of crystalline materials^12–14^. Since the 1990s, a range of surface sensitive analysis techniques, including low-energy electron diffraction (LEED)^15^, X-ray photoelectron spectroscopy (XPS)^15,16^, grazing incidence X-ray diffraction (GIXRD)^17^ and atomic force microscopy (AFM)^11,18–21^ have been applied to detect the (2 × 1) reconstruction of calcite (104). On the other hand, theoretical studies, including molecular dynamics (MD) simulations^10,22,23^ and density functional theory (DFT) calculations^11,21,24^ have been performed to search for the atomic model of this reconstructed surface and to provide insight into the origin of the (2 × 1) reconstruction. Recently, by combining high-resolution non-contact atomic force microscopy (NC-AFM) data and DFT calculations, it was established that (2 × 1) reconstruction is the most thermodynamically stable form of calcite (104)^11^.

Very recently, it has been shown that calcite (2 × 1) reconstruction can have a decisive effect on calcite adsorption properties^11,25^. With respect to the origin of the calcite (104)-(2 × 1) reconstruction, the early views proposed in the 1990s included cation ordering^26,27^. This viewpoint was recently rebutted by Rohl via DFT calculations^11^. In 2003, Rohl used MD methods and noted that an imaginary phonon mode of the unreconstructed surface signifies the necessity of surface reconstruction^22^. Nevertheless, vibrational properties obtained by MD simulations should be treated with caution since these simulations use empirical potentials^28^ and in some cases, MD methods do not yield satisfactory results for calcite^29^. Calculations based on more accurate quantum mechanical methods are thus highly desirable. Stipp also performed MD simulations and proposed that step edges may be responsible for (2 × 1) reconstruction^10^. However, the step edge effect becomes negligible for terrace sizes larger than 4.5 nm^10^ and subsequent AFM studies where cleaved surfaces were shown to be flat reported the reconstruction^11,19^. Very recently, Rahe reported that the origin of the (2 × 1) reconstruction was ‘purely thermodynamic’ via DFT simulations^11^. However, it remains unclear why the reconstructed surface has a lower total energy. In addition, it is not known whether (2 × 1) reconstruction is a spontaneous process or whether an energy barrier exists during reconstruction, i.e., the nature of reconstruction.

In the present work, we attempt to obtain a fundamental understanding of calcite (104)-(2 × 1) reconstruction by applying DFT methods. Notably, DFT simulations have recently played an important role in elucidating the mechanisms of crystal surface reconstruction^30–33^ because of their relatively high accuracy and ability to investigate the underlying electronic structure in depth. Our results unambiguously show that (2 × 1) reconstruction results from the intrinsic demands of surface atoms to increase the coordination numbers. We also calculate vibrational properties to determine whether the unreconstructed surface has imaginary phonon modes. Finally, the climbing image nudged elastic band (CI-NEB) method^34^ is applied to ascertain whether there is an energy barrier during the reconstruction.

## Results and discussion

**Fig. 1:**
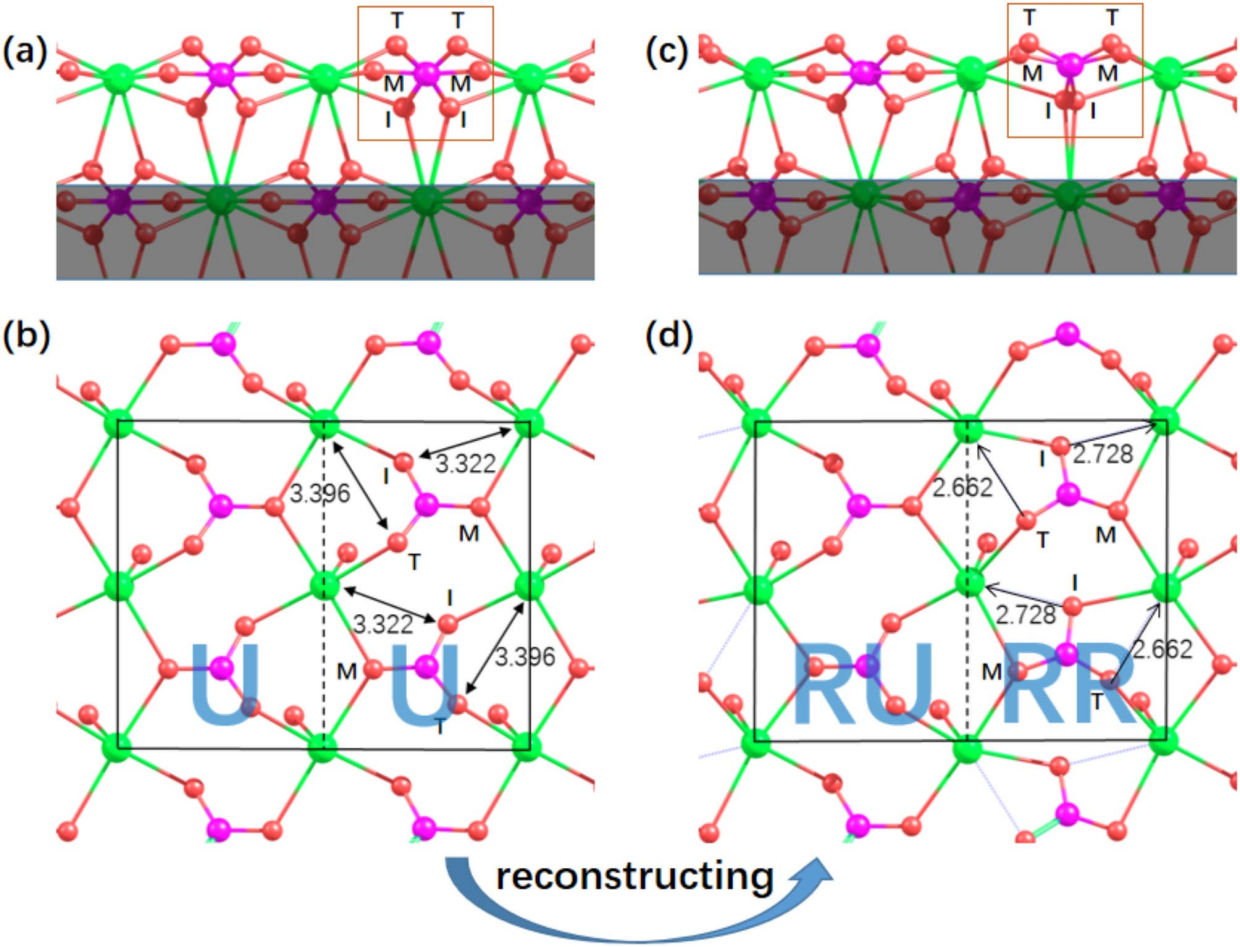
Atomic structures of unreconstructed and reconstructed calcite (104). The top and bottom panels are the side view and top view, respectively. The shaded area of the side view is not shown in the top view for clarity. The unreconstructed surface (U) is shown on the left, whereas the reconstructed surface is shown on the right. RU and RR represent the unreconstructed half and the reconstructed half of a (2 × 1) unit cell of the reconstructed surface, respectively. Color scheme: Ca in green, C in purple, and O in red. T = topmost, M = middle, I = innermost.

We first describe the structural features of both unreconstructed and reconstructed calcite (104) surfaces. The optimized calcite bulk lattice constants are a = b = 5.027 Å and c = 17.061 Å, which are in good agreement with the experimental values^35^ (4.991 and 17.062 Å). In the calcite bulk, each Ca atom is bonded to six O atoms, whereas each O atom binds with two Ca atoms. All the Ca–O bond lengths (the nearest Ca–O distance) are 2.368 Å. The next-nearest (NN) Ca–O distance is considerably greater (3.481 Å). In the top layer of the unreconstructed surface, the coordination number (CN) of each Ca atom is reduced to five (Fig. 1a, b). Each outmost O atom of one carbonate group binds with only one Ca atom, whereas the other two O atoms still bind with two Ca atoms. The (2 × 1) reconstruction features a rotation of the carbonate group within one half (called ‘the reconstructed half’, denoted by ‘**RR**’ hereafter) of each (2 × 1) unit cell, whereas structure variation in the other half (called ‘the unreconstructed half’, denoted by **RU**) is much less apparent^11,22^ (Fig. 1c, d). The atomic structure of the optimized reconstructed (104) surface is essentially the same as that obtained by Rahe^11^ since both simulations are at the same level of theory. Notably, on the basis of this structure, NC-AFM images at different tip‒sample distances have been successively reproduced^11^. Previously, a similar reconstructed structure obtained via MD simulations was shown to be able to reproduce the experimental LEED pattern^22^.

We identify that upon surface reconstruction, each surface $\:\text{C}{\text{O}}_{3}^{2-}$ group in RR forms two *additional* Ca‒O bonds. As shown in Fig. 1, each topmost O atom (T) in RR moves towards one NN Ca atom. Rotation of the $\:\text{C}{\text{O}}_{3}^{2-}$ group of approximately 18⁰ results in a significant decrease in the corresponding Ca-O distance from 3.396 Å (Fig. 1b) to 2.662 Å (Fig. 1d), i.e., a reduction of approximately 0.7 Å. Likewise, the distance between each innermost O atom (I) and one NN Ca atom substantially decreases from 3.322 Å to 2.728 Å (reduction ≈ 0.6 Å). The two Ca-O bonds (2.662 and 2.728 Å) formed upon reconstruction are approximately 0.3 Å longer than the nearest Ca-O distance in the bulk (2.368 Å) and are approximately 0.8 Å shorter than the NN Ca-O distance (3.481 Å). One may raise a question as to why previous studies have not discussed these two additional bonds (as far as the authors are aware). One reason may be that these chemical bonds are relatively long such that typical graphical programs do not show the bonding; therefore, drastic changes in Ca‒O distances were overlooked. On the other hand, all five nearest Ca–O distances associated with one $\:\text{C}{\text{O}}_{3}^{2-}$ group within the right half of the (2 × 1) cell in Fig. 1b increase upon reconstruction, but the magnitude of variation is relatively small (no greater than 0.11 Å). In the left half of the (2 × 1) cell in Fig. 1b, three of the five nearest Ca–O distances associated with one $\:\text{C}{\text{O}}_{3}^{2-}$ group increase upon reconstruction, with a maximum change of ~ 0.05 Å. The other two nearest Ca–O distances decrease slightly (a change of no greater than ~ 0.01 Å).

**Fig. 2:**
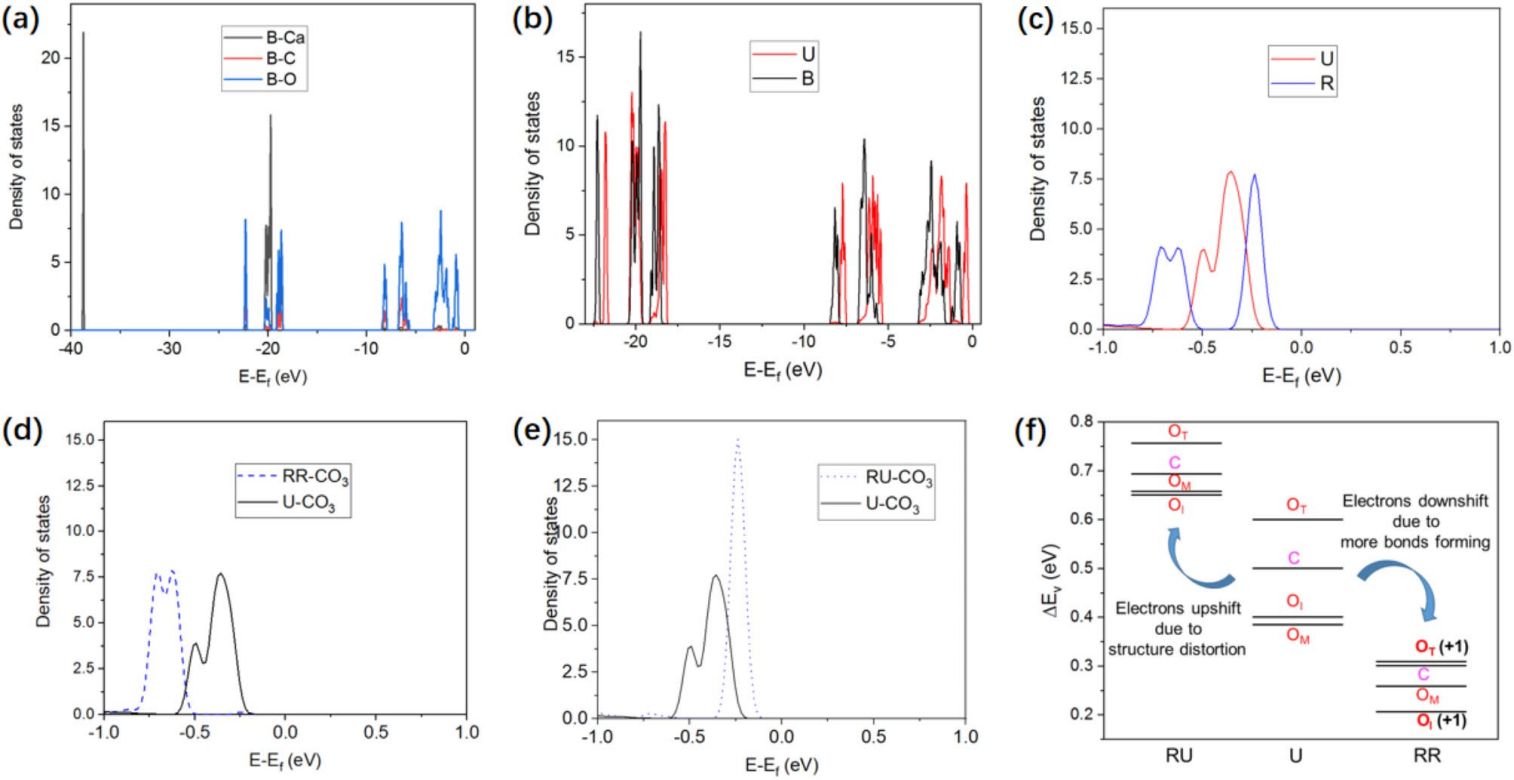
Calcite electronic structures: bulk, unreconstructed and reconstructed (104). (**a**) DOS of the calcite bulk (B) projected on different species. (**b**) DOS of the bulk and unreconstructed (U) (104). (**c**) DOS of unreconstructed and reconstructed (R) (104). (**d**) DOS associated with one carbonate group in RR (RR-CO_3_) versus the unreconstructed surface (U-CO_3_). (**e**) DOS associated with one carbonate group in RU (RU-CO_3_) versus the unreconstructed surface. (**f**) Shift in the valence electron levels associated with one carbonate group in U, RU and RR. O_T_, O_I_ and O_M_ stand for the topmost, innermost and middle oxygen atoms, respectively (see Fig. 1). ‘+1’ in the brackets means that the coordination number increases by 1.

We now discuss the electronic structures of calcite that underlie its energetics. Figure 2.a shows the density of states (DOS) of the bulk calcite. In line with the literature^36^, Ca shows high peaks at approximately − 38 and − 20 eV. DOS is dominated by C and O electrons in the energy range close to the Fermi level (-10 to 0 eV). Moreover, O has a much higher DOS than C does, as a result of the 3:1 atomic ratio and the stronger ability to attract electrons. The atomic charge is estimated via Bader analysis. In bulk calcite, the atomic charges of Ca, C and O are + 1.63, + 2.17 and − 1.27, respectively. Owing to the reduced coordination numbers, the energies of surface atoms are higher than those of their bulk counterparts. Accordingly, the DOS spectrum of the unreconstructed surface generally shifts to the right compared with that of the bulk (Fig. 2.b). For clarity, the surface DOS peak at approximately − 38 eV is not shown in Fig. 2.b, which only slightly differs from the corresponding bulk peak. On the other hand, the change in the atomic charge is small at the surface, with a maximum variation of less than 0.02 for all three species. The DOS of the surface is taken to be that associated with atoms in the top layer. We show in Supplementary Fig. 1 that the DOS of the inner layers only slightly differs from that of the bulk. Figure 2.c compares the DOS spectra of the unreconstructed and reconstructed surfaces. Notably, the DOS peaks of the reconstructed surface are typically lower and broader, reflecting the lower symmetry of the reconstructed structure. Nevertheless, the DOS spectra of both surfaces generally overlap with each other, underlying the nearly degenerate surface energies^11,24^.

Figure 1 shows that upon reconstruction, the surface forms four additional bonds per (2 × 1) unit cell. We now discuss the impact of these ‘newly formed’ bonds on electronic structures. Figure 2d shows that the DOS spectrum associated with each $\:\text{C}{\text{O}}_{3}^{2-}$ group in RR shifts to a lower energy range than that in U does. DOS below − 9 eV is not shown for clarity, which also shifts to a lower energy range. This means that the $\:\text{C}{\text{O}}_{3}^{2-}$ groups in RR are thermodynamically more stable than those in U are. In contrast, DOS spectrum of each $\:\text{C}{\text{O}}_{3}^{2-}\:$group in RU shifts to a higher energy range (Fig. 2e). To better analyse the shift in DOS, we calculate the (average) valence electron energy level $\:{E}_{v-x}$ of an atom $\:x$ via Eq. (1):

1 $$\:{E}_{v-x}=\frac{{\int\:}_{-\infty\:}^{{E}_{f}}{E}_{x}D\left({E}_{x}\right)d{E}_{x}}{{\int\:}_{-\infty\:}^{{E}_{f}}D\left({E}_{x}\right)d{E}_{x}}$$

where $\:{E}_{x}$ is the valence electron energy of atom $\:x$ and *D*$\:\left({E}_{x}\right)$ is the corresponding DOS. We then define the valence electron level shift ($\:{\varDelta\:E}_{v-x}$) as the difference between the $\:{E}_{v-x}$ of a surface atom and that of its bulk counterpart, as given by Eq. (2):

2 $$\:{\varDelta\:E}_{v-x}={E}_{v-x}\left(\text{s}\text{u}\text{r}\text{f}\text{a}\text{c}\text{e}\right)-{E}_{v-x}\left(\text{b}\text{u}\text{l}\text{k}\right)$$

As shown in Fig. 2f, at the unreconstructed surface, the topmost oxygen atoms (O_T_) have the highest $\:{\varDelta\:E}_{v}$ as a result of CN reduction (from two to one). Upon reconstruction, in RR, each outmost oxygen atom (O_T_) and each innermost oxygen atom (O_I_) form one additional bond with Ca (Fig. 1). O_T_ and O_I_ in RR exhibit noticeable decreases in the valence electron energy, with changes in $\:{\varDelta\:E}_{v}$ of -0.29 and − 0.19 eV, respectively (Fig. 2f). As a result, the difference between $\:{\varDelta\:E}_{v-{O}_{T}}$ and $\:{\varDelta\:E}_{v-C}$ is much smaller in RR than in U. Regarding O_I_, in RR, $\:{\varDelta\:E}_{v-{O}_{I}}$ becomes lower than $\:{\varDelta\:E}_{v-{O}_{M}}$, whereas in U, the contrary is true. For C and O_M_, although CNs do not change during reconstruction, hybridization between the carbon and oxygen states within each $\:\text{C}{\text{O}}_{3}^{2-}\:$group (Fig. 2.a) renders a downshift of the corresponding electronic states in RR (Fig. 2f). On the other hand, the Ca‒O bond lengths in RU mainly increase (as discussed above). Accordingly, the electron energies associated with C and O in RU increase (Fig. 2e, f).

We also investigated surface Ca atoms, each of which forms two additional bonds with oxygen upon reconstruction. The $\:{\varDelta\:E}_{v-Ca}$ of the reconstructed surface is 0.15 eV lower than that of the unreconstructed surface. The less significant variation in Ca electron energy may be related to the lower lying of Ca electron states (Fig. 2a), which are therefore less sensitive to chemical environment changes. In summary, the electron energies of all the C and O atoms decrease in RR as a result of the increase in CN and hybridization between the oxygen and carbon states. Ca electrons also shift down because CN increases, although the change is relatively small. In contrast, the electron energies of all the C and O atoms shift up in RU because of the elongation of the Ca-O bonds. Therefore, an increase in the coordination number is the key to stabilizing the reconstructed surface, which decreases the electron energies of surface Ca atoms and the electron energies of C/O atoms in RR.

**Fig. 3:**
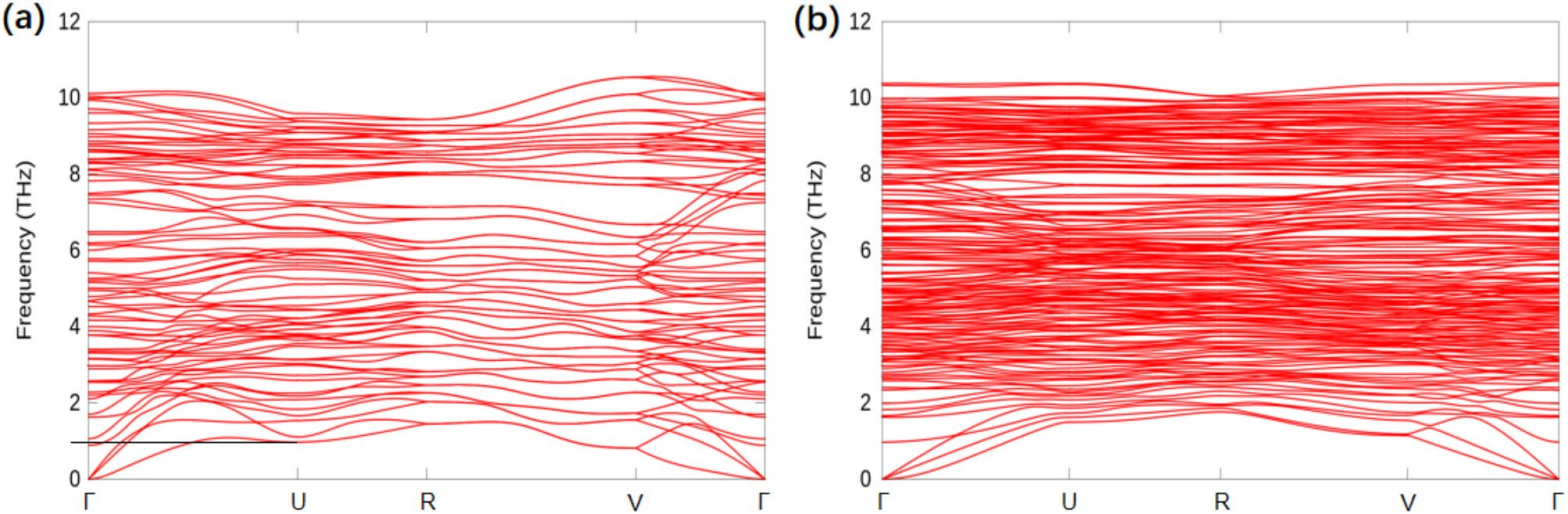
Vibrational properties of calcite (104). (**a**) Phonon spectrum of the unreconstructed surface. (**b**) Phonon spectrum of the reconstructed surface. Special points in the two-dimensional Brillouin zone: Γ(0,0), U(0.5,0), R(0.5,0.5) and V(0, 0.5).

By performing MD simulations, Rohl et al.. reported an imaginary phonon mode for the unreconstructed surface and suggested that (2 × 1) reconstruction was a spontaneous process^22^. However, in a more recent study, Magdans et al.. reported an unreconstructed (104) surface under both dry and humid atmospheric conditions via GIXRD^17^. This signals the stability of the unreconstructed surface; therefore, one would expect an energy barrier separating the unreconstructed and the reconstructed states. To address this problem, we have calculated phonon spectra for both the unreconstructed and the reconstructed (104), as shown in Fig. 3. It is apparent that there are more bands in the phonon spectrum of the reconstructed surface as a result of the symmetry reduction. Rohl reported that the imaginary phonon mode of the unreconstructed surface is located at (0.5,0) in the Brillouin zone^22^, which is the U point in Fig. 3. However, our DFT simulations show that although U is a local minimum of the lowest phonon band for the unreconstructed surface, the corresponding frequency is positive (~ 0.9 THz). In both panels of Fig. 3, no imaginary phonon mode exists. Consequently, our study reveals that both the unreconstructed and reconstructed surfaces are dynamically stable. We have also calculated the phonon spectrum of the unreconstructed surface by applying the PBE functional and the DFT-D3 method with Becke‒Johnson damping^37,38^. The results show that there is no imaginary phonon mode associated with the unreconstructed surface, irrespective of the method applied within the DFT framework. Therefore, our DFT results support Magdans’s work, which reported an unreconstructed (104) surface under both dry and humid conditions^17^.

**Fig. 4:**
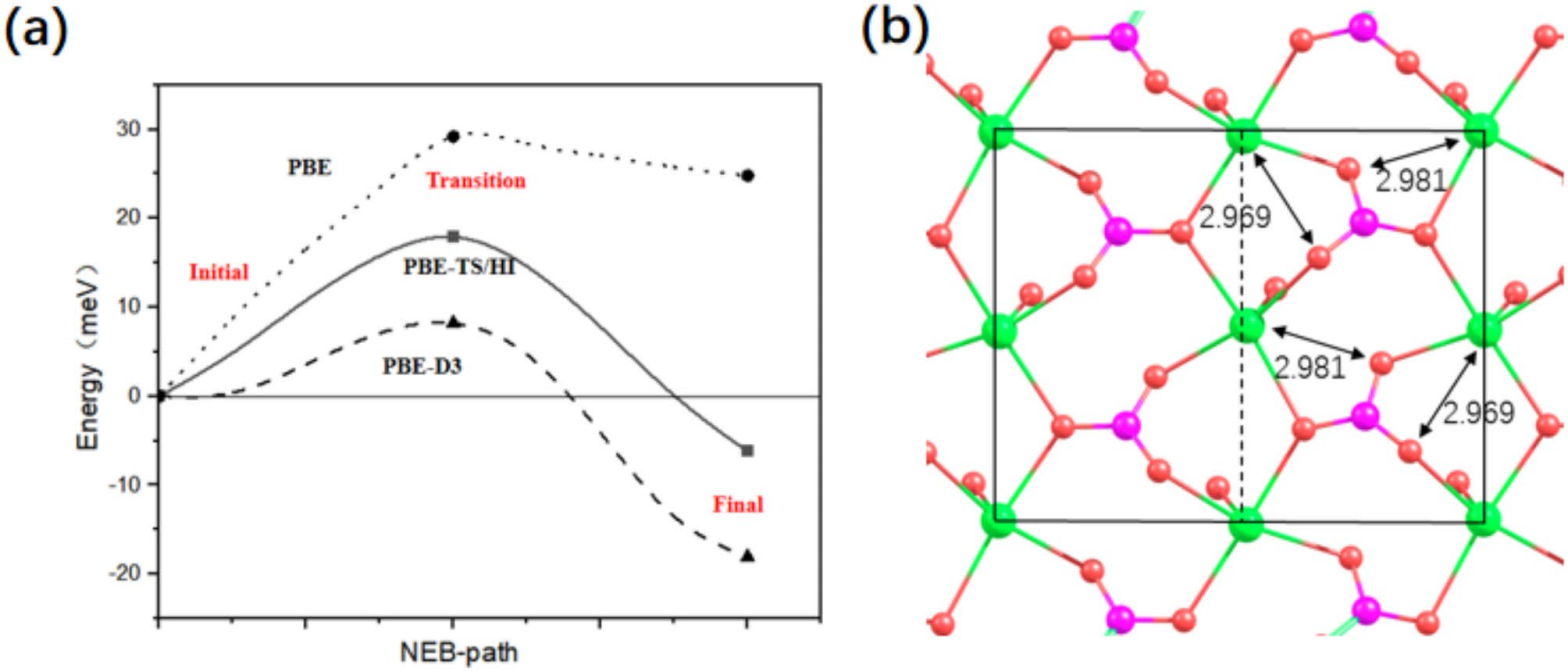
Probing the possible energy barrier during calcite (104) reconstruction. (**a**) Minimum energy path calculated via the CI-NEB method. ‘Initial’, ‘Transition’ and ‘Final’ represent the initial state (unreconstructed surface), the transition state, and the final state (reconstructed surface), respectively. (**b**) Atomic structure of TS.

We have also applied the CI-NEB method^34^ to probe the possible energy barrier. As shown in Fig. 4a, there appears to be a single energy barrier between the two states. Figure 4b shows that in the transition state, carbonate groups rotate approximately 10⁰ in the right half of the (2 × 1) unit cell, whereas they rotate 18⁰ in the final reconstructed state. The two Ca‒O distances corresponding to the two ‘newly formed’ Ca‒O bonds when fully reconstructed are 2.969 and 2.981 Å, respectively. These values are approximately 0.6 Å greater than the nearest Ca-O distance in the bulk (2.368 Å) and are approximately 0.5 Å smaller than the NN Ca-O distance (3.481 Å). Therefore, the transition state can be understood as a state where these two additional Ca–O bonds are about to be formed. The activation energy (E_a_) is 14 meV per (2 × 1) unit cell for the formation of the reconstructed surface. Conversely, E_a_ for the transition from a reconstructed surface to an unreconstructed surface is 20 meV per unit cell. Considering that the calculated activation energy is rather small, we also apply the PBE functional without vdW corrections and the DFT-D3 method with Becke‒Johnson damping^37,38^ to check this. In line with the literature^11,24^, while both DFT methods with van der Waals interactions predict that the reconstructed surface is slightly favoured, the semilocal PBE functional predicts that the unreconstructed surface is favoured. Nevertheless, Fig. 4a shows that there is a nonvanishing but low energy barrier between the unreconstructed surface and the reconstructed surface, irrespective of the DFT method used.

## Summary

In summary, by applying DFT methods with van der Waals corrections, we unambiguously show that calcite (104)-(2 × 1) reconstruction is driven by the demand of surface atoms to increase the coordination number. At the surface, four additional Ca‒O bonds are formed per (2 × 1) unit cell upon reconstruction as a result of carbonate group rotation. Within the surface layer, the electron energies associated with carbonate groups in RR and Ca atoms are effectively lowered because new bonds form. In contrast to Rohl’s work^22,]^ which performed MD simulations, our DFT phonon spectra indicate that both the unreconstructed and the reconstructed surfaces are stable. The low energy barrier obtained by the CI-NEB method suggests that the detected surface structure should be critically dependent on the experimental conditions. That is, both the unreconstructed and the reconstructed surfaces are expected to be observable under certain experimental conditions since they are either metastable or stable states. Very recently, it has been shown that calcite (2 × 1) reconstruction can have a decisive effect on calcite adsorption properties^11,21,25^. We believe that our findings can significantly advance the understanding of the surface-related phenomena of calcite.

## Methods

All calculations are performed via the Vienna ab-initio simulation package (VASP)^39^ projector-augmented wave (PAW) potentials^40^. Most of the calculations are based on the PBE^41^ functional with the Tkatchenko–Scheffler method with iterative Hirshfeld partitioning^42^. We also use the PBE functional without vdW corrections and the PBE-D3 method in NEB calculations to make comparisons. In line with Rahe’s work^11^, van der Waals forces play a decisive role in forming the (2 × 1) structure. When the semilocal PBE functional is used, the total energy (per (2 × 1) unit cell) of the unreconstructed surface is lower than that of the reconstructed surface by 25 meV. On the other hand, when the Tkatchenko–Scheffler method is applied, the total energy of the reconstructed surface is lower than that of the unreconstructed surface by 6 meV. Plane-wave cutoff energy is set to 500 eV, and the convergence of energy is 10^− 6^ eV. The 5 × 8 × 5 and 2 × 3 × 1 Monkhorst–Pack k-point meshes are set to calculate the calcite bulk and (104), respectively. Atomic and electronic structure analyses are based on 7-layer fully optimized structures, although we have found that a 4-layer model is thick enough to obtain the converged surface energy (in line with Rahe’s work^11^). Phonon analyses are performed via the 4-layer model (including 12 atomic layers). Phonon spectra are calculated via the finite difference approach. In NEB calculations, we include nine images between the initial state and the final state to start the simulations.

## Electronic supplementary material

Below is the link to the electronic supplementary material.

Supplementary Material 1

## Data Availability

Data is provided within the manuscript or supplementary information files.
