# Supplementary material for: Formation mechanism of the (2 × 1) reconstruction of calcite (104)
Source: Sci Rep. 2025 Apr 8;15:11988. doi: 10.1038/s41598-025-95955-2 (PMC11978872; doi:10.1038/s41598-025-95955-2)
Supplement: Supplementary file 1 — Supplementary Material 1 [file 41598_2025_95955_MOESM1_ESM.pdf]

Supplementary Information

Formation mechanism of the (2 × 1) reconstruction of calcite (104)

Haojun Zhou[1], Yingquan Chen[1], Mingyue Ding[2] and Xiaoliang Zhong[1,*]

[1]School of Energy and Power Engineering, Huazhong University of Science and Technology, Wuhan 430074, China
[2]School of Power and Mechanical Engineering, Wuhan University, Wuhan 430072, China.

e-mail: xzhong@hust.edu.cn

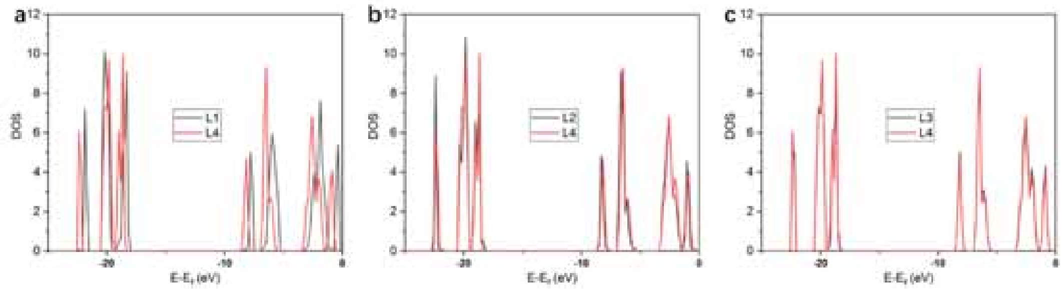

Supplementary Figure 1. Layer-by-layer density of states of unreconstructed calcite (104) surface based on a 7-layer model. L1, L2, L3 and L4 denotes the topmost, the second, the third and the fourth layer, respectively.

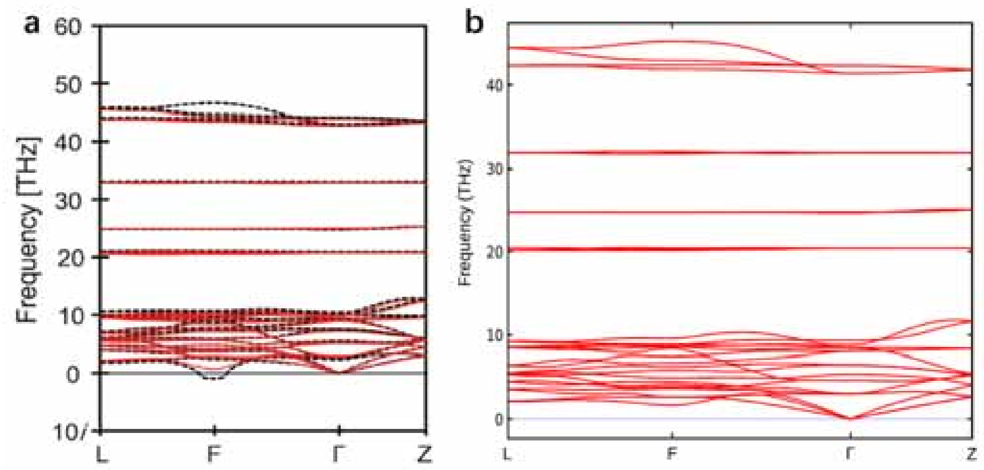

Supplementary Figure 2. Comparison of calcite bulk phonon spectrums calculated by different functionals. a Phonon spectrums of bulk calcite as given by Ukita using the LDA functional (J. Appl. Phys. 120, 142118). The red and black lines are phonon dispersions under normal atmospheric pressure and under 2 GPa, respectively. **b** Phonon spectrum of bulk calcite using the Tkatchenko-Scheffler method with iterative Hirshfeld partitioning.

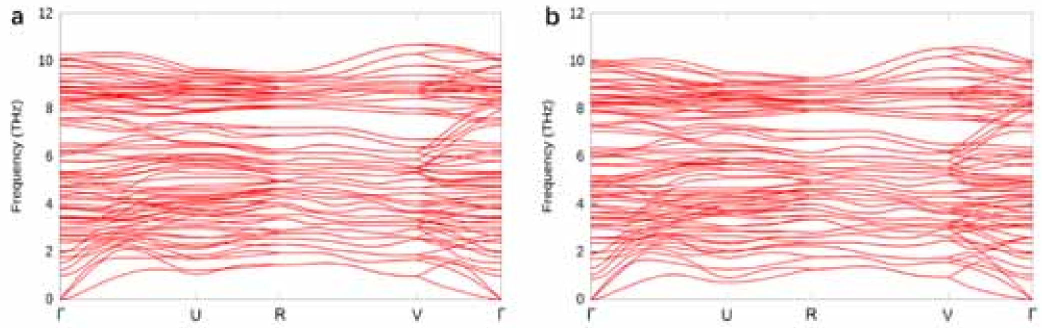

Supplementary Figure 3. Comparison of calcite (104) surface phonon spectrums calculated by different functionals. a Phonon spectrum calculated by the DFT-D3 method with Becke-Johnson damping. **b** Phonon spectrum calculated by PBE functional.
